# Fucoidan Extracted from *Fucus Evanescens* Prevents Endotoxin-Induced Damage in a Mouse Model of Endotoxemia

**DOI:** 10.3390/md12020886

**Published:** 2014-01-31

**Authors:** Tatyana A. Kuznetsova, Natalya N. Besednova, Larisa M. Somova, Natalya G. Plekhova

**Affiliations:** 1 Laboratory of Immunology, G.P. Somov Research Institute of Epidemiology and Microbiology, Siberian Branch of Russian Academy of Medical Sciences, 1 Selskya St., Vladivostok 690087, Russia; E-Mail: besednoff_lev@mail.ru; 2 Laboratory of Pathomorphology, G.P. Somov Research Institute of Epidemiology and Microbiology, Siberian Branch of Russian Academy of Medical Sciences, 1 Selskya St., Vladivostok 690087, Russia; E-Mails: l_somova@mail.ru (L.M.S.); pl_nat@hotmail.com (N.G.P.)

**Keywords:** sulfated polysaccharide, fucoidans, immunity, cytokines, hemostasis, endotoxin, lipopolysaccharides

## Abstract

An important problem of treating patients with endotoxemia is to find drugs to reduce the negative effects of endotoxin on the organism. We tested fucoidan (sulfated polysaccharide) from the brown alga *Fucus evanescens* as a potential drug in a mouse model of endotoxemia inducted by lipopolysaccharide (LPS). The survival time of mice injected with LPS increased under fucoidan treatment compared with the group of mice injected with LPS only. The preventive administration of fucoidan to mice with endotoxemia resulted in inhibition of increased levels of proinflammatory cytokines (TNFα and IL-6), as well as decreasing of the processes of hypercoagulability. The parenteral or *per os* administration of fucoidan resulted in decreasing the degree of microcirculatory disorders and secondary dystrophic-destructive changes in parenchymal organs of mice with endotoxemia. Taken together, these results demonstrate that fucoidan prevents endotoxin-induced damage in a mouse model of endotoxemia and increases the mice’s resistance to LPS.

## 1. Introduction

Fucoidans are sulfated branched heteropolysaccharides from cell walls of brown algae. Fucoidans are non-toxic substances possessing diverse pharmacological activities including the action on innate and adaptive immunity as well as anti-inflammatory, antitumor, antioxidant, anticoagulant, *etc.* [1,2,3,4,5,6,7,8]. These activities are of great interest for the clinical use of fucoidans. In particular, there is great potential for fucoidan application in the treatment of sepsis and endoxemia for alleviating endotoxin-induced damage. It is based on their anti-inflammatory and antioxidant properties, the ability to provide cytoprotective effects on dendritic cells as important effectors of innate immunity [3,4,6,8].

Currently, sepsis remains one of the most urgent problems of modern medicine. This is due to the steady upward trend in the incidence of sepsis in the “post-antibiotic” era and high mortality despite intensive research on its treatment and prevention. Induced by endotoxin of Gram-negative bacteria, sepsis is of particular interest because of the central role of endotoxin in various manifestations of septic shock. Endotoxin or lipopolysaccharide (LPS), a component of the cell membrane of Gram-negative bacteria, acts as a trigger in sepsis [9]. There is a concept of the endotoxin playing an aggressive role in immunopathogenesis of various infectious and noninfectious diseases. According to this concept, endotoxin’s aggressive role is a universal general biological process due to the redundancy of LPS in the bloodstream and the absolute or relative lack of antiendotoxin immunity [10,11]. When getting into the systemic circulation, LPS (endotoxin) interacts with the humoral and cellular immune factors and triggers numerous pathological, including cardio-pulmonary and vascular dysfunction, hypercoagulability and Disseminated Intravascular Coagulation (DIC-syndrome), acute renal and hepatic failure and other disorders. These processes are the result of both the direct and indirect effects of endotoxin [11,12].

There has been little scientific research dealing with fucoidan application in experimental endotoxemia. For example, according to a previous report, the survival time of the mice injected with a lethal dose of *E. coli* LPS increased upon condition preventive administration of fucoidan from *Fucus vesiculosis*. Upon investigating the mechanisms, the authors found that fucoidan had a cytoprotective effect, contributing to recovery of the population of dendritic cells and increased expression of the molecules Bcl-2, Bcl-xL, cIAP-1 in splenocytes of the endotoxemic mice [13].

In this study we tested fucoidan from brown alga *Fucus evanescens* as a drug for reducing endotoxin negative effects in mouse model of endotoxemia. This fucoidan shows positive effects on macroorganisms, including immunomodulatory, pro/antiinflammatory, antitumor, anticoagulant, *etc.* [14,15,16,17].

## 2. Results and Discussion

### 2.1. Effects of Fucoidan on Survival Times in Mouse Model of Endotoxemia

In mice injected with LPS (group I) the following toxic symptoms were observed 1 h after: weakness (lethargy), shortness of breath, refusal of food and water; wool of mice became wet and disheveled. Two hours afterwards, the body temperature increased and diarrhea commenced. These signs are the evidence of a systemic inflammatory response due to the launch of a pro-inflammatory cytokines cascade and other inflammation mediators [18,19,20,21]. In 3–4 h, clinical symptoms of intoxication reached the maximum, then paralysis and paresis of the limbs developed. By the end of the first day to the beginning of the second day, all mice of this group were dead.

**Figure 1:**
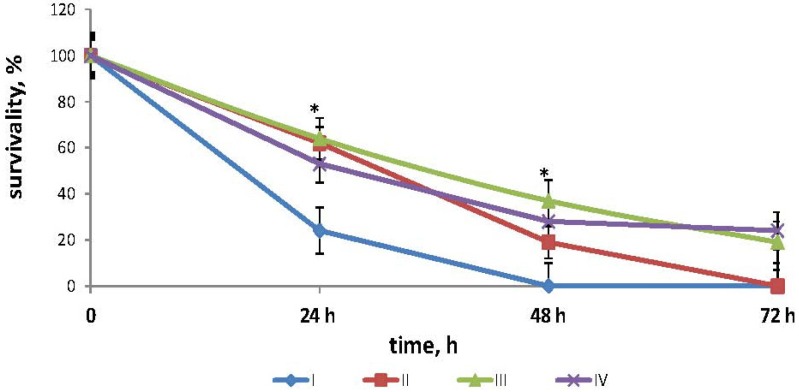
Effects of fucoidan on survival times in mouse model of endotoxemia. Date represents the mean ± S.D. *n* = 15. group I—mice, injected intraperitoneally with LPS in dose LD_100_; group II—mice, injected with lipopolysaccharide (LPS) in dose LD_100_ and then treated with fucoidan in dose 5 mg/kg (medical scheme: 3 times a day subcutaneously); group III—mice, treated with fucoidan (preventive scheme: for 10 days daily subcutaneously) and then injected with LPS in dose LD_100_; group IV—mice, treated with fucoidan in dose 50 mg/kg *per os* for 3 weeks daily and then injected with LPS in dose LD_100_; *—the significance level of differences between III and I; IV and group I(*p* <0.05) (*z* ≥ 2021).

The effects of fucoidan on the survival times in mouse model of endotoxemia are shown in Figure 1. The mean lifespan (MLS) of the mice injected with LPS in dose LD_100_ (positive control group I) was 27.1 ± 2.2 h. In the mice injected with LPS and then treated with fucoidan in dose 5 mg/kg (medical scheme: 3-times a day subcutaneous (group II), MLS was 43.2 ± 3.4 h (*z* = 1145, *p* = 0.693 compared with the group I), and all the animals also died. In the mice treated with fucoidan (preventive scheme: daily for 10 days subcutaneous) and then injected with LPS (group III), MLS increased to 52.8 ± 4.3 h (*z* = 2040, *p* = 0.047 compared with the group I), the survival after 72 h was 18.9 ± 1.2%. In the mice treated with fucoidan in dose 50 mg/kg *per os* daily for 3 weeks and then injected with LPS (group IV) MLS increased to 57.6 ± 3.6 h, the survival was 24.4 ± 4.3% (*z* = 2240, *p* = 0.042 compared with the control group I).

### 2.2. Effects of Fucoidan on Cytokines Production in Mouse Model of Endotoxemia

**Figure 2:**
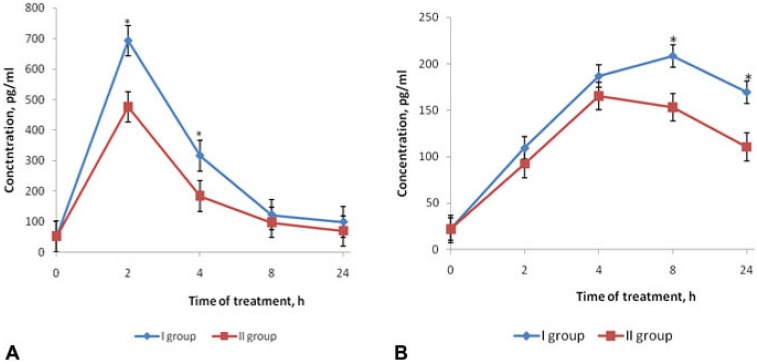
Effects of fucoidan on the dynamics of TNFα (**A**) and IL-6 (**B**) levels in blood serum of BALB/c mice in mouse model of endotoxemia. Date represents the mean ± S.D. *n* = 6; group I—mice, injected intraperitoneally with LPS in dose LD_100_; group II—mice, treated with fucoidan (preventive scheme: daily for 10 days subcutaneously) and then injected with LPS in dose LD_100_; * *p* < 0.05—I group compared to the II. Mann-Whitney test.

The effects of fucoidan on the dynamic of proinflammatory cytokines TNFα (A) and IL-6 (B) in blood serum of BALB/c mice are shown in Figure 2. Evaluation of the cytokine level showed a significant elevation of TNFα concentrations in serum in 2 h after LPS injection (group I) compared with its initial level (0 h) (Figure 2A). Within 4 h, the concentration of this cytokine decreased and within 24 h, it gradually approached the initial level (0 h). In mice of group II, which had received preventive fucoidan treatment, the concentration of TNFα at the peak of its production (2–4 h) were lower than in group I (*p* < 0.05) (Figure 2A). The corrective effect of fucoidan on the TNFα level in the early stages of endotoxemia (2–4 h) is particularly important, as this period corresponds to the maximum of clinical manifestations of endotoxemia.

The IL-6 dynamics showed a different pattern compared to the TNFα level in mice of group I: the IL-6 level in blood serum also exceeded the initial values (0 h), but the peak levels were recorded after 8 h, then the values gradually reduced to 24 h. Under fucoidan’s influence (group II) within 8–24 h, the IL-6 level was significantly lower compared with that in mice of group I (*p* < 0.05) (Figure 2B).

### 2.3. Effects of Fucoidan on Hemostasis Parameters in Mouse Model of Endotoxemia

Results of the hemostasis parameters of mice are shown in Table 1. These results indicate that the experimental endotoxemia (group I) led to development of marked hypercoagulability in the early stages (4 h after LPS injection). Such results manifested themselves by increase of the total plasma coagulant potential (accelerated blood clotting in basic clotting tests—activated partial thromboplastin time (APTT), thrombin time (TT), prothrombin time (PT) and inhibition of fibrinolytic activity (FA) compared to the control (group IV). These signs are characteristic of the initial stage of the Disseminated Intravascular Coagulation (DIC-syndrome) [22,23,24]. In mice treated with fucoidan according to the therapeutic scheme (group II) hypercoagulation was relieved, which is supported by the APTT- and TT-tests and the FA activation, but the blood clotting characteristics did not return to the reference levels in the control (group IV). Preventive fucoidan treatment (group III) induced hypocoagulation shifts, manifesting themselves by prolonged blood clotting time compared to the LPS control (group I) and to the negative control (group IV) (*p* < 0.05). These processes were associated with higher FA levels (*p* < 0.05) and recovery of fibrinogen (FG) levels (*p* < 0.05) compared to the group I, which approached the values of the control (group IV) (Table 1). Presumably, such effects of fucoidan are due to anticoagulant and fibrinolytic activities [14,15].

**Table 1:** Effects of fucoidan on hemostasis parameters of mice 4 h after injection of LPS. Date represents the mean ± S.D. *n* = 5. Mann-Whitney test.

Hemostasis parameters	Group of mice			
	LPS (group I)	Fucoidan (group II)	Fucoidan (group III)	Control (0.85% NaCl) (group IV)
APTT (s)	25.0 ± 3.9	38.5 ± 3.9	75.6 ± 13.5	47.4 ± 5.7 *p* (IV–I) < 0.05
		*p* (II–I) < 0.05	*p* (III–I) < 0.05	
		*p* (II–IV) > 0.05	*p* (III–IV) < 0.05	
PT (s)	11.6 ± 1.8	14.0 ± 1.8	23.2 ± 4.8	16.8 ± 5.3 *p* (IV–I) > 0.05
		*p* (II–I) > 0.05	*p* (III–I) < 0.05	
		*p* (II–IV) > 0.05	*p* (III–IV) >0.05	
TT (s)	12.8 ± 0.8	15.7 ± 2.0	67.8 ± 9.8	18.6 ± 1.2 *p* (IV–I) < 0.05
		*p* (II–I) < 0.05	*p* (III–I) < 0.05	
		*p* (II–IV) < 0.05	*p* (III–IV) < 0.05	
FA (min)	533 ± 56	430 ± 58	350 ± 61	310 ± 65 *p* (IV–I) < 0.05
		*p* (II–I) < 0.05	*p* (III–I) < 0.05	
		*p* (II–IV) < 0.05	*p* (III–IV) > 0.05	
FG (g/L)	6.1 ± 0.9	4.8 ± 0.45	4.4 ± 0.42	4.1 ± 0.52 *p* (IV–I) < 0.05
		*p* (II–I) < 0.05	*p* (III–I) < 0.05	
		*p* (II–IV) > 0.05	*p* (III–IV) > 0.05	

Comparison of the dynamics clotting parameter and levels serum cytokine (TNFα) in mice injected with LPS showed, that hyperproduction of these cytokines preceded the increasing of hypercoagulation. This is in line with the assumption key role of the proinflammatory cytokines (specifically, thereof hyperproduction) in hypercoagulation and fibrinolysis inhibition in sepsis and endotoxin shock [22,25,26]. By the end of the 1st day after LPS injection we observed increasing of blood clotting time, activation FA and drop FG level, reflecting the development of hypocoagulation changes. By this time, the TNFα concentrations reduced significantly, indicating the attenuation of LPS-induced cytokine cascade.

Treatment of mice with *F. evanescens* fucoidan promoted a reduction of hypercoagulation and inhibition of elevated TNFα and IL-6 levels. The latter is particularly important in the mechanisms of fucoidan action, because the therapeutic strategy of sepsis and endotoxin shock treatment is directed against the hyperinflammatory cascade elements, specifically—against these cytokines. By realizing these mechanisms, fucoidan promotes attenuation of the clinical manifestations of endotoxicosis, reduction of hypercoagulation symptoms and other signs of the DIC syndrome, and as a result promotes an increase of resistance to LPS toxicity.

In their experimental studies, a number of authors demonstrated that anticoagulants (heparin, lepirudin, hirudin) also lead to reduction of hypercoagulability and to inhibition of proinflammatory cytokine cascade during LPS-induced endotoxemia [22,24,27,28].

### 2.4. Effects of Fucoidan on Histopathological Changes in Target Organs of Mice during Endotoxemia

As presented above, microcirculation disorders, hypoxia and severe dysfunction of organs and body systems, multiple organ failure (MOF) are signs of endotoxemia. MOF is characterized by lesions in various organs and tissues with predominance of organ failure (pulmonary, cardiac, renal, hepatobiliary, *etc.*) symptoms. Without adequate treatment, these symptoms steadily progress and become fatal [19,21,28,29].

**Figure 3:**
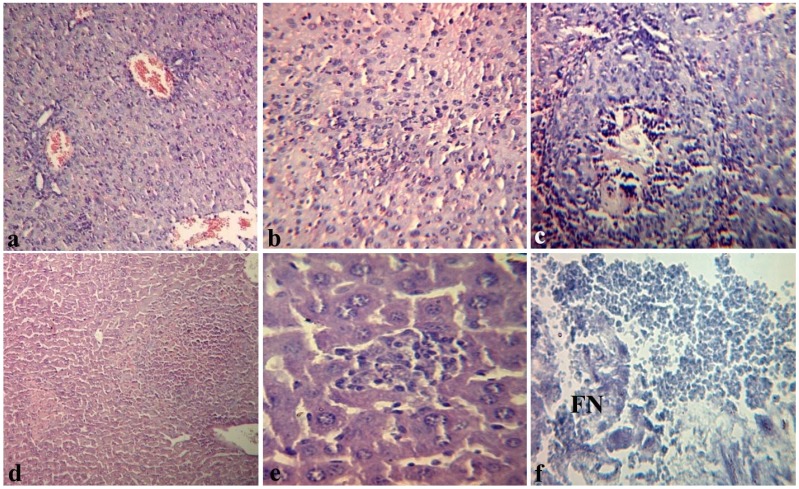
Histopathological changes in the liver of mice 4 (**a**,**b**,**c**) and 24 h (**d**,**e**,**f**) after intraperitoneal injection of *Y. pseudotuberculosis* LPS in dose LD_100_ (group I): (**a**) marked hyperemia of portal tract vessel with swelling and local destruction of its wall × 100; (**b**) necrobiosis of hepatocytes with polymorphocellular infiltration. 200×; (**c**) typical pseudotuberculosis foci with central zone necrosis. 200×; (**d**) necrotic foci with karyorrhexis. 100×; (**e**) polymorphocellular granuloma. 200×. Hematoxylin eosin stain (**a**–**e**); (**f**) the area of fibrinoid necrosis (FN). 200×. Shueninov stain.

We conducted the histopathological study of “target organs” or “shock organs” (liver, kidneys, lungs, heart) during endotoxemia. For example, Figure 3 illustrates changes in liver of mice. After 4 h, we observe hyperemia mainly of central veins (terminal hepatic venules) and vessels of portal tracts with the presence of local destructive changes of the vascular wall in the liver of mice injected with LPS (group I). We observed eritrostasis mixed with polymorphonuclear leukocytes in the lumen of blood vessels. In some vessels there were aggregations of erythrocytes and fibrin with plasma separation and formation of a homogeneous eosinophilic mass (Figure 3a). In the hepatic parenchyma we observed necrobiosis of hepatocytes with polymorphnonuclear infiltration (Figure 3b); a few necrotic foci were met near the vessels (often centrilobular, sometimes in the form of typical pseudotuberculosis foci with karyorrhexis in the center) (Figure 3c). After 8–17 h post-infection the LPS-induced damage in the liver was increased; 24 h after the LPS inoculation, alongside the vascular changes, we found the typical pseudotuberculosis necrotic foci with fine detritus in the center (Figure 3d), numerous small granulomas without karyorhexis of the central zone (Figure 3e), fibrinoid necrosis (FN) of portal tracts’ vascular walls and perivascular inflammatory reaction (Figure 3f).

Over the course of our observations, the histopathological changes in the kidneys, heart and lungs were characterized by microcirculatory disorders with development of vascular walls’ fibrinoid swelling and destructive inflammatory changes in parenchyma. In 24 h, we observed necrotic changes in these organs in the microvascular walls with conglomerates in their lumen, which stained positively for fibrin.

Therefore, we observed histopathological manifestations of the LPS-induced endotoxemia including the “sludge-phenomenon” (irreversible aggregation) of erythrocytes in vessels, hemocirculatory disorders, the presence of microthrombs, the increased permeability and destruction of the vascular endothelium, and dystrophic-necrotic changes in the cells of parenchymal organs, which were signs of DIC. These changes grow in proportion to the duration of the pathological process, caused by microcirculation disorder and by development of secondary disorder of organs blood circulation, and possibly primary lesions due to direct toxic effects of LPS.

Similar data, illustrating the LPS-induced damage in target organs, is shown in several studies [30,31,32].

**Figure 4:**
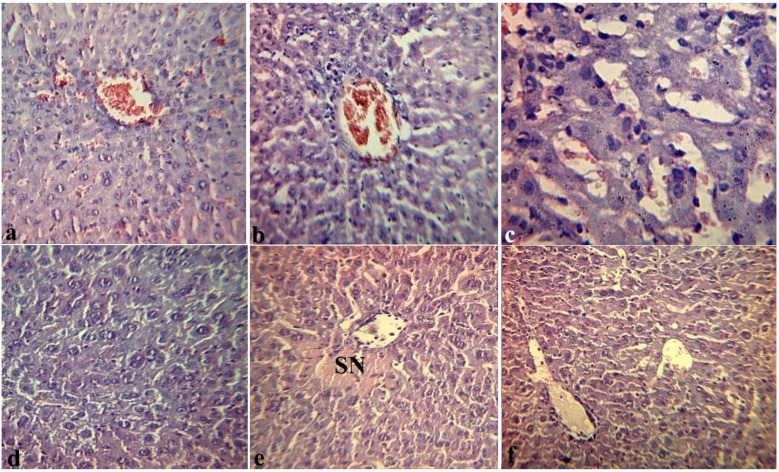
Effects of fucoidan on histopathological changes in the liver of mice 4 h (**a**,**b**,**c**) and 24 hours (**d**,**e**,**f**) after intraperitoneal injection of *Y. pseudotuberculosis* LPS in dose LD_100_ (group II): (**a**) structure of vascular endothelium and hepatocytes is preserved, erythrocytes in the lumen of the sinusoidal capillaries. 200×; (**b**) the loose polymorphocellular infiltration around the hepatic vessels. 200×; (**c**) granular degeneration of hepatocytes, the site of necrobiosis. 200×; (**d**) area of necrobiosis in hepatocytes with desorganisation of parenchyma. 200×; (**e**) sphenoid necrosis (SN) around the central vein of the hepatic lobule. 200×; (**f**) eosinophilic mass and sporadic leucocytes in the lumen of the vessels. 200×. Hematoxylin eosin stain (**a**–**f**).

Figure 4 presents the effects of fucoidan on histopathological changes in the liver of mice that received fucoidan (preventive scheme: for 10 days daily subcutaneous) and then LPS (group II). Over 4 h, in the liver of mice we observed moderate expansion of intralobular and interlobular vessels without significant plethora. Destructive changes in the vascular endothelium and hepatocytes were not observed.

The lumen of blood vessels was filled with erythrocytes and/or eosinophilic mass, sometimes mixed with single leukocytes. The endothelial lining of blood vessels is not broken; the eritrodiapedetic and plazmorrea was not observed. Only in a few vessels the focal endothelial destruction without formation of diapedetic hemorrhages around them was observed. However, the sinusoidal capillaries of hepatic lobules were populated by erythrocytes (Figure 4a). Around some vessels we observed the loose polymorphocellular infiltration, mainly by mononuclear and Kupffer cells (Figure 4b). In the liver lobules around of the central vein we often observed the area of modified hepatocytes with granular or eosinophilic dystrophy, and, to a lesser extent, with necrobiosis (Figure 4c). 

In the liver and in other target-organs of mice that received fucoidan, distinct vascular endothelial injuries were not observed. Over 24 h, the intensity of pathological changes in target organs of the mice in the group II was also significantly less than in mice of the group I, that did not receive fucoidan: we observed areas of necrobiosis in hepatocytes with desorganisation of parenchyma (Figure 4d) and sphenoid necrosis (SF) around the central vein of the hepatic lobule (Figure 4e), eosinophilic mass and isolated leucocytes in the lumen of the vessel (Figure 4f).

These microcirculatory disturbances are residual signs of DIC.

Thus, the results of these studies have shown that clinical and morphological manifestations of endotoxemia induced by intraperitoneal injection of bacterial LPS were less pronounced in the mice that received fucoidan. So, we revealed a normalizing effect of fucoidan on the state of parenhymal organs of mice with endotoxemia.

## 3. Experimental Section

### 3.1. Animals

In experiments we used 184 male BALB/c mice (20–22 g) and 200 outbred male mice from Stolbovaya Breeding Center. The animals were handled in accordance with the European Convention for Protection of Vertebrate Animals used in Experimental and other Studies. Cets No.: 123 Strasbourg, 18.03.1986.

### 3.2. Reagents

Isolation and characterization of Fucoidan was performed by researches of Laboratory of Enzyme Chemistry, G.B. Elyakov Pacific Institute of Bioorganic Chemistry, Far-Eastern Branch, Russian Academy of Sciences, 159 100-Let Vladivostoku Ave, Vladivostok, 690022, Russian Federation, supervisor Prof. Tatyana N. Zvyagintseva.

Fucoidan is a sulfated polysaccharide—1→3;1→4-α-l-fucan with molecular weight of 20–40 kDa. Its monosaccharide composition includes fucose, galactose, xylose, glucose in ratio 71:9:10:8, the ratio of fucose and sulfate residue is 1:0.8 [33]. Fucoidan was isolated from brown algae *Fucus evanescens* of Okhotsk Sea by the hot extraction method [33].

Lipopolysaccharide (LPS) was isolated from a strain of 598 IB *Yersinia pseudotuberculosis* by Westphal method [34] in Laboratory of Carbohydrates and Lipids Chemistry, G.B. Elyakov Pacific Institute of Bioorganic Chemistry, Far Eastern Branch, Russian Academy of Sciences, 159 100-Let Vladivostoku Ave, Vladivostok 690022, Russian Federation, supervisor Prof. Nadegda A. Komandrova, kindly provided by Dr. Raisa P. Gorshkova.

### 3.3. Model of Endotoxemia

Model of experimental endotoxemia was producing by intraperitoneal administration of LPS in dose LD_100_ (6.25 ± 0.5 mg/kg) to BALB/c mice or to outbread mice. Observation for the animals was carried out during 120 h.

### 3.4. The Survival Time of Mice

The protective effect of fucoidan was evaluated by the percent of survivality (%) and mean lifespan (MLS) of outbred mice injected with *Yersinia pseudotuberculosis* LPS in dose LD_100_. The percent of survival was calculated according to the formula: (number of surviving mice/total number of surviving and dead mice) × 100%; the mean lifespan (MLS) was calculated according to the formula: total lifespan of all mice/number of mice in the group.

Sixty BALB/c mice were randomized into 4 groups (*n* = 15 in each group): I-mice were injected intraperitoneal with LPS in dose LD_100_; II-mice were injected with LPS in dose LD_100_ and then treated with fucoidan in dose 5 mg/kg (medical scheme: 3 times a day subcutaneous); III-mice were treated with fucoidan (preventive scheme: for 10 days daily subcutaneous) and then injected with LPS in dose LD_100_; IV-mice were treated with fucoidan in dose 50 mg/kg *per os* for 3 weeks daily and then injected with LPS in dose LD_100_.

### 3.5. Determining the Level of Cytokines in the Serum of Mice

The serum levels of TNFα and IL-6 in BALB/c mice were measured by ELISA kits (BD Biosciences OptEIATM Set Mouse; USA) according to the instructions supplied with the kits. The results were recorded in terms of the optical density measured at 450 nm with Multiscan RC (Labsystems; Finland). Seventy two BALB/c mice were randomized into 3 groups: group I was included 30 mice injected intraperitoneal with LPS in dose LD_100_; group II was included 30 mice treated with fucoidan (preventive scheme: for 10 days daily subcutaneous) and then injected with LPS in dose LD_100_. III control group was included 12 mice injected with 0.85% NaCl. The level of cytokines in the serum of mice were determinated in 0, 2, 4, 8, 24 h after inoculation of LPS, *n* = 6.

### 3.6. Determination of Hemostasis Parameters

The coagulation component of hemostasis was evaluated by the activated partial thromboplastin time (APTT), prothrombin time (PT), and thrombin time (TT) of blood clotting; fibrinogen (FG) level was measured, fibrinolytic activity (FA) was evaluated in the clot spontaneous euglobin lysis test with reagents from firm (Technology Standard) (Russia).

Two hundred outbread mice were randomized into 4 groups: group I—mice were injected intraperitoneal with LPS in dose LD_100_; group II—mice were injected with LPS in dose LD_100_ and then treated with fucoidan in dose 5 mg/kg (medical scheme: 3 times a day subcutaneous); group III—mice were treated with fucoidan (preventive scheme: for 10 days daily subcutaneous) and then LPS in dose LD_100_; group IV—mice were injected with 0.85% NaCl (control). Parameters of hemostasis were determinated in 4 h after LPS injection in blood pool from 10 mice; the study was repeated 5-fold, *n* = 5.

### 3.7. Histopathological Studies

Sixty four BALB/c mice were randomized into 4 groups: group I—mice were injected intraperitoneal with LPS in dose LD_100_; group II—mice were treated with fucoidan (preventive scheme: for 10 days daily subcutaneous) and then LPS; group III—mice were treated with fucoidan *per os* in dose 50 mg/kg for 3 weeks daily and then LPS; group IV—mice were injected with 0.85% NaCl (control). The mice were anesthetized in 4, 8, 17 and 24 h after LPS inoculation (*n* = 4). Liver, kidneys, lungs, hearts samples were fixed in 10% neutral buffered formalin solution. The material handling and paraffin embedding were performed with standard methods; sections of 5–6 mm were stained with hematoxylin-eosin and for detection of fibrin—according to Shueninov method. Observation and photomicrography were performed with a «Zeiss» microscope (Germany).

### 3.8. Statistical Analysis

Statistical analysis was performed using the Statistica 7.0 software package (StatSoft, Tulsa, OK, USA) by Mann-Whitney test for independent samples. To compare survival rates of the two groups of animals the log-rank *z*-test (with Yates correction) was used.

The data are expressed as the mean ± S.D.; *p*-values < 0.05 was considered statistically significant.

## 4. Conclusions

We tested the effects of fucoidan extracted from the brown alga *Fucus evanescens* on endotoxin-induced damage in mouse model of endotoxemia. The results of these studies demonstrate that preventive parenteral or *per os* fucoidan administration increases the survival times of mice, leads to inhibition the increasing levels of proinflammatory cytokines (TNFα, IL-6), to attenuation of the hypercoagulation and microcirculatory disorders, secondary dystrophic-destructive changes in the liver, kidneys, lungs and hearts of mice. Furthermore, the results indicate that fucoidan effectively regulates the immunity and hemostasis systems in experimental endotoxemia, attenuates the course of the DIC syndrome, prevents endotoxin-induced damage in mouse model of endotoxemia and, in the long term, has the potential for development a drug for reducing the negative effects of endotoxin.
